# Insertional effect following electrode implantation: an underreported but important phenomenon

**DOI:** 10.1093/braincomms/fcae093

**Published:** 2024-03-28

**Authors:** Clement Hamani, Benjamin Davidson, Nir Lipsman, Agessandro Abrahao, Sean M Nestor, Jennifer S Rabin, Peter Giacobbe, Rosana L Pagano, Ana Carolina P Campos

**Affiliations:** Sunnybrook Research Institute, Toronto, ON M4N 3M5, Canada; Harquail Centre for Neuromodulation, Sunnybrook Health Sciences Centre, Toronto, ON M4N 3M5, Canada; Division of Neurosurgery, Sunnybrook Health Sciences Centre, University of Toronto, Toronto, ON M4N 3M5, Canada; Sunnybrook Research Institute, Toronto, ON M4N 3M5, Canada; Harquail Centre for Neuromodulation, Sunnybrook Health Sciences Centre, Toronto, ON M4N 3M5, Canada; Division of Neurosurgery, Sunnybrook Health Sciences Centre, University of Toronto, Toronto, ON M4N 3M5, Canada; Sunnybrook Research Institute, Toronto, ON M4N 3M5, Canada; Harquail Centre for Neuromodulation, Sunnybrook Health Sciences Centre, Toronto, ON M4N 3M5, Canada; Division of Neurosurgery, Sunnybrook Health Sciences Centre, University of Toronto, Toronto, ON M4N 3M5, Canada; Sunnybrook Research Institute, Toronto, ON M4N 3M5, Canada; Harquail Centre for Neuromodulation, Sunnybrook Health Sciences Centre, Toronto, ON M4N 3M5, Canada; Division of Neurology, Department of Medicine, Sunnybrook Health Sciences Centre, University of Toronto, Toronto, ON M4N 3M5, Canada; Sunnybrook Research Institute, Toronto, ON M4N 3M5, Canada; Harquail Centre for Neuromodulation, Sunnybrook Health Sciences Centre, Toronto, ON M4N 3M5, Canada; Department of Psychiatry, Sunnybrook Health Sciences Centre, University of Toronto, Toronto, ON M4N 3M5, Canada; Sunnybrook Research Institute, Toronto, ON M4N 3M5, Canada; Harquail Centre for Neuromodulation, Sunnybrook Health Sciences Centre, Toronto, ON M4N 3M5, Canada; Division of Neurology, Department of Medicine, Sunnybrook Health Sciences Centre, University of Toronto, Toronto, ON M4N 3M5, Canada; Rehabilitation Sciences Institute, University of Toronto, Toronto M5G 1V7, Canada; Sunnybrook Research Institute, Toronto, ON M4N 3M5, Canada; Harquail Centre for Neuromodulation, Sunnybrook Health Sciences Centre, Toronto, ON M4N 3M5, Canada; Department of Psychiatry, Sunnybrook Health Sciences Centre, University of Toronto, Toronto, ON M4N 3M5, Canada; Laboratory of Neuroscience, Hospital Sírio-Libanês, São Paulo, SP CEP 01308-060, Brazil; Sunnybrook Research Institute, Toronto, ON M4N 3M5, Canada; Laboratory of Neuroscience, Hospital Sírio-Libanês, São Paulo, SP CEP 01308-060, Brazil

**Keywords:** insertional effect, electrode, deep brain stimulation, microthalamotomy, glia

## Abstract

Deep brain stimulation has revolutionized the treatment of movement disorders and is gaining momentum in the treatment of several other neuropsychiatric disorders. In almost all applications of this therapy, the insertion of electrodes into the target has been shown to induce some degree of clinical improvement prior to stimulation onset. Disregarding this phenomenon, commonly referred to as ‘insertional effect’, can lead to biased results in clinical trials, as patients receiving sham stimulation may still experience some degree of symptom amelioration. Similar to the clinical scenario, an improvement in behavioural performance following electrode implantation has also been reported in preclinical models. From a neurohistopathologic perspective, the insertion of electrodes into the brain causes an initial trauma and inflammatory response, the activation of astrocytes, a focal release of gliotransmitters, the hyperexcitability of neurons in the vicinity of the implants, as well as neuroplastic and circuitry changes at a distance from the target. Taken together, it would appear that electrode insertion is not an inert process, but rather triggers a cascade of biological processes, and, as such, should be considered alongside the active delivery of stimulation as an active part of the deep brain stimulation therapy.

## Teaser

Hamani *et al*. examine how the insertion of deep brain stimulation electrodes into the brain has been shown to induce clinical benefits prior to stimulation onset. They review cellular, circuitry and behavioural changes associated with the electrode insertion and the impact of this effect on clinical trials.

## Graphical Abstract

**Graphical Abstract:**
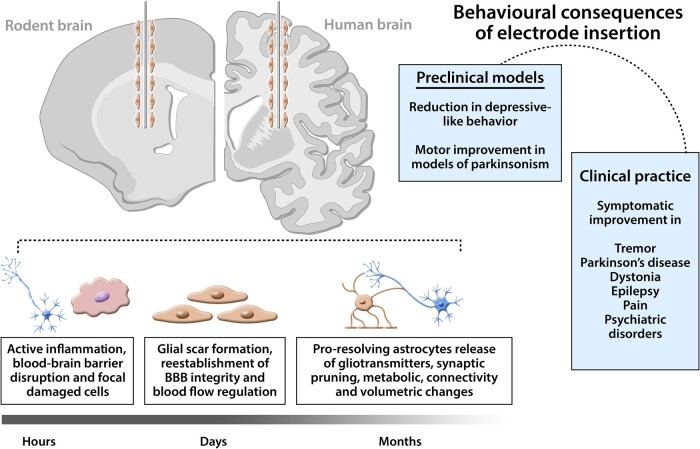

## Introduction

Since its introduction, deep brain stimulation (DBS) has become a standard of care in the treatment of movement disorders and epilepsy and is under investigation for various neuropsychiatric disorders.^1-4^ After electrodes are implanted, clinicians often wait 2–4 weeks before activating the DBS system. Interestingly, during the initial days or weeks following surgery, patients frequently experience a noticeable clinical improvement prior to the delivery of stimulation. First reported in conditions such as tremor and Parkinson’s disease,^5-7^ this response has also been documented in patients treated for chronic pain,^8^ epilepsy^9^ and even psychiatric disorders.^10^ Because the clinical effects of electrode insertion are often similar to those observed after ablative procedures, this phenomenon was originally thought to be caused by a focal ‘microlesion’. However, decades of clinical observations and preclinical study have demonstrated that the clinical response associated with the ‘insertional effect’ may be due to complex biological processes rather than merely tissue disruption.

In preclinical models, the insertion of electrodes has been shown to induce substantial behavioural improvements, often mirroring what is observed in humans. Preclinical models allow for histopathologic examination at various timepoints after insertion, which has led to several explanatory mechanisms being proposed, including inflammatory responses near the electrode site, the activation of astrocytes, a focal activation hyperexcitability of neurons and the development of neuroplastic changes.

This review aims to characterize the insertional effect from four different angles. First, we describe the neuropathological changes that occur following the implantation of brain electrodes and probes. Second, we present some of the behavioural effects and mechanisms responsible for the development of an insertional effect in animal models. Third, we report the clinical consequences of electrode insertion. Finally, we outline how the insertional effect may interfere with the results of clinical trials.

### Neurohistopathology of the insertional effect

Most neurohistopathological studies to date have been conducted in naïve rodent brains following the insertion of electrodes and microdialysis probes. The latter is discussed as it may help to characterize neurochemical tissue responses to brain implants.

### Initial trauma related to the implant

**Figure 1:**
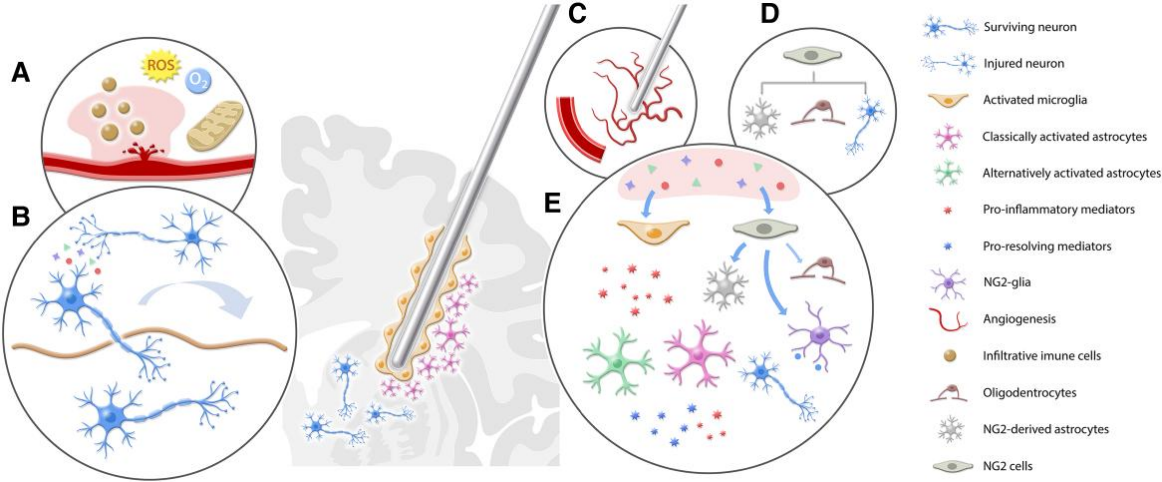
**Neurochemical mechanisms of electrode insertion in the brain parenchyma.** (**A**) The mechanical insertion of the electrode into the target leads to a disruption of small blood vessels causing local edema, focal bleeding and leakage from the blood brain barrier. Injury to the brain parenchyma may lead to mitochondrial stress and the release of reactive-oxygen species (ROS) that may be responsible for a transient decrease in local oxygenation. (**B**) This initial disruption may induce a transient decrease in local glucose metabolism, a functional disruption in connectivity between the target region and interconnected structures, as well as neuronal stress. (**C**) In response to the insult, pericytes and endothelial cells will attempt to counter the acute microenvironment stress by re-establishing the BBB integrity, attenuating the infiltration of peripheral immune cells, and inducing angiogenesis. (**D**) Resident NG2-precursor cells migrate to the implanted site and differentiate into astrocytes, oligodendrocytes or neurons in the attempt to mitigate the insult response. (**E**) The stress induced by the electrode implantation leads to the formation of several damage-associated molecular patterns (DAMPs) that are responsible for the chemoattraction of classically activated microglia and astrocytes. These cells form a protective capsule, commonly called ‘glial scar’, that leads to local metabolic and functional changes due to the release of pro-inflammatory and pro-resolving mediators. These mediators may influence the maturation of NG2-precursor cells into astrocytes and axonal-growth neurons that will influence the connectivity of the target area.

The introduction of probes and electrodes into the brain leads to a series of short- and long-term changes resultant from the interaction between the biomaterial and the surrounding neural tissue.^11-18^ At first, the insertion of an implant focally damages cells, capillaries, the extracellular matrix, and disrupts the blood brain barrier (BBB) (Fig. 1).^15,19,20^ Injured capillaries may lead to leakage of erythrocytes, activation of platelets^15^ and the development of a localized haematoma.^21^ BBB disruption may culminate with the extravasation of inflammatory plasma proteins, a decrease in focal oxygen/nutrient delivery, microglial activation, mitochondrial dysfunction, increased oxidative stress and the accumulation of neurotoxic products in the brain parenchyma.^20,22-25^

Hours after the initial injury, a transient focal reduction in glucose metabolism can be observed surrounding the electrode,^26^ and in areas anatomically connected with the lesioned site.^27^ Other early responses include microglia activation and migration towards the implant,^28,29,30-32^ as well as the activation of local astrocytes that undergo morphological changes, becoming hypertrophied (Fig. 1).^13,26,30,33-35^

Within a few days, an encapsulating sheath begins to form around the electrode.^28,29,36^ Pericytes, present in capillary walls, undergo morphological changes and contribute to local angiogenesis within the newly forming sheath.^37^ This vascularized sheath helps to re-establish the BBB integrity, blood flow regulation and to reduce brain exposure to peripheral inflammatory cells and pathogens.^37-39^

The abovementioned processes lead to neuronal apoptosis and local cell death along the tract and at the target site (Fig. 1).^19^ Also injured are fibres *en passant*, which may project to nearby or distant brain regions.^40^ Argyrophilic neurons and fine silver deposits denoting degenerating neurites, nerve terminals and fibres may be observed as early as 24 h after probe implantation, becoming increasingly prominent with longer survival times.^40^ However, not all surrounding neurons perish. Surviving neurons may show an increased expression of immediate early genes^41^ and potentially release excitatory amino acids in response to the implant-induced injury.^42-44^ The vitality of neurons surrounding the electrodes is essential for a stable signal transmission and eventual stimulation responses.^45^

### Glial response and reactive gliosis

Over the first six weeks following electrode insertion, the surrounding tissue undergoes a process known as reactive gliosis, a response of the CNS to seal the damaged site, restrict focal inflammatory responses and limit neuronal loss.^34,46-51^ Reactive gliosis involves the recruitment of astrocytes, microglia and NG2-expressing glial precursors,^48,52,53^ as well as angiogenesis and revascularization of the area surrounding the lesion (Fig. 1).^54^ Within the first week after the insertion of brain electrodes or probes, a large number of reactive astrocytes, microglia and laminin labelled vessels may be observed around the implant.^55^ Between 2 and 6 weeks, connective tissue and a loosely organized sheath of glial cells may be encountered.^13,49,51,56-62^ This process has been demonstrated in rodents,^46-49,63^ cats^64^ and non-human primates.^65^

In addition to helping form an encapsulating sheath, astrocytes play a crucial role in neurotransmitter clearance, neuroplasticity, neuroinflammation, the regulation of cerebral blood flow and the maintenance of extracellular ionic concentrations.^66-69^ Following the insertion of electrodes/probes into the brain, glia cells in the vicinity of the implants are activated and undergo morphological changes (Fig. 1).^19,20^

Reactive astrocytes are characterized by hypertrophic somata and processes,^70^ synthesize a higher than normal amount of glial-fibrillary acidic protein (GFAP) and express progenitor markers, such as vimentin, nestin and synemin.^48,71,72^ When active, a large proportion of astrocytes assume an A1 pro-inflammatory secretion phenotype, losing their ability to prune synapses and regulate glutamatergic clearance.^73^ Astrocytes may also undergo an A2 anti-inflammatory/restorer phenotype, which is associated with neuroprotective and reparative effects via the secretion of neurotrophic factors and synaptogenesis.^73-75^ Mature astrocytes do not normally divide, but may acquire stem cell properties and generate cells that initiate repair after brain injury.^46^

Microglia also contribute to the formation of a physical barrier between the device and brain parenchyma.^19^ Under normal circumstances, microglia exist in a resting or ramified state near blood vessels, constantly monitoring the environment for pathological or injury signals.^76-78^ Upon electrode insertion, they are attracted to the injured territory, and extend lamellipodia to help ensheath the implants. These cells are stimulated following exposure to pathogen- or endogen-associated molecular patterns, endogenous ligands that act on toll-like-receptors (e.g. heat-shock proteins and fibrinogen), or when there is a lack of immunosuppressive signals.^77^ Activated microglia undergo structural changes, assume an amoeboid morphology,^77,79^ proliferate,^80^ and migrate to the site of injury in an attempt to enclose the pathological process and shield the BBB (Fig. 1).^77,78,81,82^ In contrast to the resting state, active microglia secrete chemokine attractants (e.g. monocyte chemoattractant proteins and macrophage inflammatory proteins), pro-inflammatory cytokines (e.g. tumour necrosis factor-α, certain interleukins and interferons), transforming growth factors, reactive oxygen and nitrogen species (ROS, RNS) and neurotrophic factors (e.g. brain derived neurotropic factor, nerve growth factor and neurotrophin-3).^15,19,83,84^ The immune response promoted by microglia is critical to restrict damage and facilitate repair, helping to limit toxicity and eliminate foreign materials and debris.

NG2 cells are resident glial progenitors that exist in the developing and mature mammalian CNS.^85-88^ NG2 cells are primarily the precursors of oligodendrocytes, but can also form other glial and neuronal cell types.^85^ In addition to their role as glial progenitors, these cells migrate, proliferate and inhibit axonal growth, being an important element in the formation of glial scars.^89,90^ Both NG2 glia and microglia migrate towards inserted probes (Fig. 1). NG2 glia, however, act in a more protracted manner (within hours), forming synaptic connections with nearby neurons.^91^ When neurons are injured by the insertion of implants, the differentiation of NG2 glial cells into astrocytes to form the glial scar reduces the number of cells that may differentiate into oligodendrocytes.^91^ This may yield deficits in the remyelination of injured neurons, contributing to the neuronal apoptosis observed following a surge in focal inflammatory processes in the injured tissue.^90,91^

### Chronic implants

**Figure 2:**
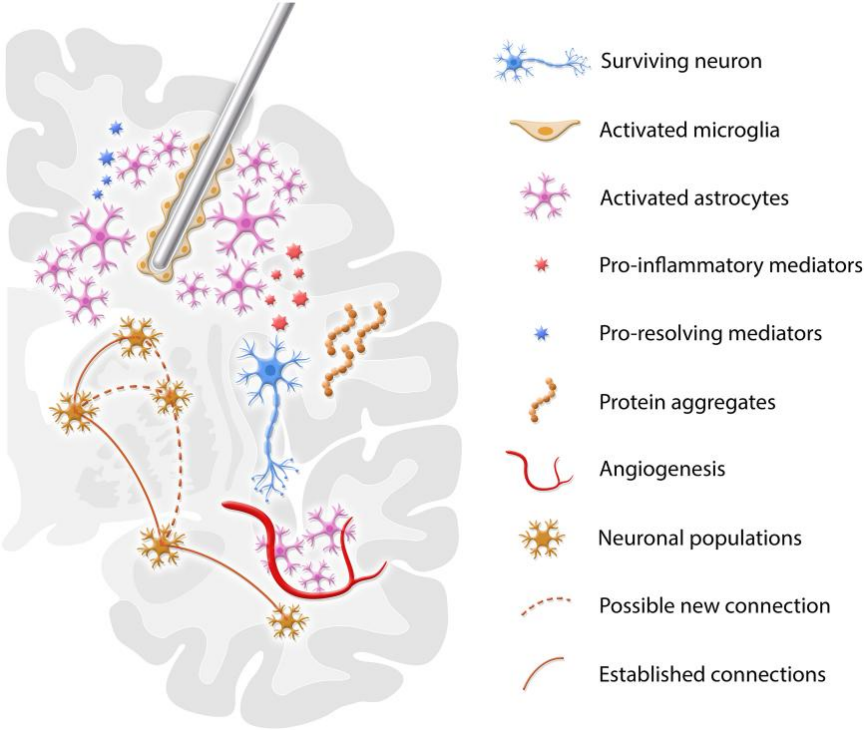
**Mechanisms of electrode implantation.** Preclinical studies suggest that classically activated microglia and astrocytes may play a pivotal role in the mechanisms of electrode implantation, since anti-inflammatory drugs attenuate its antidepressant-like effect. Classically activated microglia may also be responsible for the clearance of local protein aggregates. On the other hand, glial scar astrocytes are often related to an alternative activation that releases pro-resolving mediators and increases glutamate clearance in an attempt to promote homoeostasis and is associated with the development of hyperexcitability in surviving neurons. Electrode implantation is also responsible for the increased expression of colocalized serotoninergic receptors and p11 (S100A10). This response is influenced by inflammatory mediators and may affect the increased input to efferent areas. Beyond the modulation of previously existing connections, electrode implantation may interfere with the formation of new connections with surrounding areas. Efferent and distant regions are also affected, presenting an increase in tissue volume, accounted by angiogenesis and astrocytes.

By 6–12 weeks post-electrode or probe implantation, reactive astrocytes are largely condensed in an increasingly compact sheath (Fig. 2).^55,58^ The continuous presence of the implant results in a sustained response that maintains the gliotic sheath.^41,51,58^ The capsule that forms around the implant is surrounded by an extracellular matrix.^30,92-94^ Tight junctions between microglia and astrocytes during scar tissue formation may act as a biochemical and mechanical barrier. Should the implant be a microdialysis probe or a neurochemical sensor, this may prevent the diffusion of ions, solutes and chemical signalling molecules secreted from surrounding neurons. Over time, the activity of microglia and the acute foreign body reaction to the implant may fade, despite the presence of the sheath.^95^

Compared to the normal tissue, the immediate vicinity of the implants tends to have a lower nerve fibre density and a reduced number of cell bodies.^45,96-98^ That said, neurons near the gliotic process display increased activity, with reduced inhibitory synaptic currents, a downregulation of glutamine synthetase and reduced gamma-aminobutyric acid transmission.^99^ In addition, astrocytes can release gliotransmitters, form intricate functional connections with neurons and modulate both pre- and postsynaptic transmission (Fig. 2).^20,49,100-102,99^ Evidence of altered neuronal function can be found in regions projecting to or receiving projections from the target. These include volumetric changes, an increase in hippocampal neurogenesis, altered circuit connectivity, increased metabolic activity and blood vessel diameter (Fig. 2).^57,103-105^ This suggests that the chronic presence of electrodes in the brain parenchyma is not an inert event. Astrocytes and other glial elements change the normal functioning of nearby neurons, leading to a series of consequences, both focally and at a distance from the target.

While many of the findings above have been studied in rodents, similar observations have been noted in non-human primates, including reactive gliosis, the accumulation of microglia, the development of a glial sheath and some degree of neuronal loss.^65,106,107^

### Human implants

#### General considerations

To investigate the tissue response to the chronic implantation of leads in humans, post-mortem samples from patients treated with DBS have been analysed. Data from such studies, however, need to be considered with caution, as samples are collected from patients with different pathological states, contrasting with preclinical material obtained from naïve animals. Moreover, individuals who undergo post-mortem autopsy have usually been treated with DBS for prolonged periods of time. As a result, disentangling the consequences of electrode implantation and stimulation may not always be possible.

#### Neuropathology

Similar to preclinical work, most human reports revealed long-standing gliosis, with the presence of Rosenthal fibres (accumulation of aggregated glial-fibrillary acidic protein in astrocytes), as well as gemistocytic and fibrillary astrocytes around the electrodes.^59,108-120^ Studies described the presence of a fibrous gliotic sheath around the electrode site with a thickness that varied from 25 μm to over 150 μm.^108,119^ Additional cells in the vicinity of the implants were microglia, mononuclear leucocytes, macrophages and multinucleated giant cells.^59,111,112,116-119,121,122^ Collagenous accumulation has also been reported.^108^

In contrast to preclinical studies, neuronal loss did not seem to be a prominent feature in human samples.^113,119^ Tissue damage surrounding the electrodes has been characterized by vacuolization, myelin loss, axonal spheroids, iron deposits (uncommon) and spongiosis (rare).^121^ However, some of these responses were only described when electrodes composed of materials other than platinum/iridium were used,^119^ and in older studies, which did not consider the safety limits of charge and pulse amplitude.^119,123-125^ Most recent pathological studies did not reveal neuronal loss at a distance > 500 μm from the electrode site.^59,126^ In addition to the material of the implants, differences in neuronal death between human and animal studies may be due to the timing of electrode removal. In preclinical work, electrodes are removed right after death or even in live animals. This may injure local cytoarchitecture,^57^ as nearby glial cells may adhere to the implant.^122^ In pathological human specimens, the post-mortem removal of the implants occurs long after death in patients who had their electrodes implanted for a long period of time. As a result, the surrounding glial sheath is often compact and only a few cells are adherent to the implant.^58^

To disentangle the neuropathological consequences of electrode implantation and chronic stimulation, a strategy adopted by a few studies was to compare tissue samples surrounding active (i.e. used to deliver chronic stimulation) and non-active stimulation contacts. In general, while both showed similar levels of gliosis,^115,121,127^ enlarged axons and axonal spheroids were more often noticed around active contacts.^121^ Despite these small differences, based on the similar findings noted in the two scenarios, it is likely that the histopathological response to DBS described in recent studies might be largely related to the implants, and not the delivery of current *per se*.

## Preclinical models: behavioural effects of electrode insertion and mechanisms

Despite the vast clinical use of DBS for the treatment of movement disorders, the behavioural consequences of electrode insertion have been mainly documented in preclinical models of psychiatric and cognitive disorders. The most convincing behavioural effects came from studies comparing rodents who either underwent surgery without electrode implantation or received electrode implants with or without stimulation. Another study design in which the insertional effect was appreciated involves the use of serial behavioural measurements before and after surgery (i.e. at baseline, following electrode implantation and after the delivery of stimulation). This design, however, does not allow for the measurement of long-term consequences of electrode insertion, since DBS is often introduced days/weeks after the surgical procedure.

When considering the insertional effect in rodents, one should take into account the ratio between electrode diameter and brain volume. Though electrodes commonly used in humans are ∼5–15-fold thicker than the ones used in rodents, the human brain volume is orders of magnitude larger. This difference is maximal in frontal cortical regions, but is also pronounced in subcortical structures. Despite this limitation, preclinical models have been instrumental to develop mechanistic studies and to support the notion that the insertional effect has a biological substrate. As observed in humans, the behavioural effects of electrode insertion in rodents were often similar to those observed following DBS administration, albeit of lesser magnitude.^128,129^

### Behavioural data

In patients with treatment refractory depression, DBS delivered to the subgenual cingulum has been investigated with promising results.^10,128,130,131^ To study potential mechanisms and the kinetics of DBS, stimulation has been applied to an analogous region (i.e. ventromedial prefrontal cortex; vmPFC) in rodents undergoing different behavioural paradigms.^104,128-130,132-136^ The placement of vmPFC electrodes in the absence of stimulation was found to produce antidepressant-, anxiolytic- and antianhedonic-like effects in the forced swim test, novelty suppressed feeding and sucrose preference test.^104,128-130,133-136^ In some of these studies, the effects of electrode insertion were long-lasting^133^ and had a magnitude comparable to that observed after DBS.^104,128-130,133-136^

In models of cognitive degeneration, contrasting results have been reported. A significant improvement in the radial arm water maze test was found in Alzheimer’s disease transgenic animals briefly implanted with hippocampal electrodes.^137^ In contrast, no major differences in memory performance were found between rats that did or did not receive nucleus basalis of Meynert electrode implants in the 192 IgG-saporin model.^138^

In humans, DBS delivered to the subthalamic nucleus (STN) is considered to be a standard of care treatment for motor symptoms in Parkinson’s disease.^139,140^ To study mechanisms of STN DBS in preclinical models, naïve and parkinsonian rodents implanted with electrodes were tested in different motor and cognitive paradigms. In naïve rodents undergoing a serial reaction time task, electrode insertion increased motor time, while low current DBS reduced premature responses.^141^ In addition, rats with implanted electrodes had a significant memory impairment in the novel object recognition task.^82^ As for motor effects, both electrode insertion and stimulation delivered to hemiparkinsonian rodents reduced immobility time on a bar,^142,143^ while only the latter treatment improved amphetamine-induced rotations^142-144^ and motor performance in the rotarod test.^144^ In summary, STN electrode insertion was found to play a role on impulsivity and memory disturbance, while it was only associated with motor improvement in some tests. In contrast, the insertion of electrodes in the pedunculopontine nucleus led to a mild exacerbation in freezing of gait.^145^

### Mechanisms

Though the mechanisms responsible for the insertional effect are largely unknown, preclinical studies have considered a few hypotheses. The first is the development of a neuroinflammatory response to the injury produced by the electrodes and a subsequent glial reaction.^82^ As described above, the continuous presence of brain implants results in a sustained response that maintains a gliotic sheath, which is largely comprised of reactive astrocytes and microglia.^41,51,58^ The systemic administration of anti-inflammatories, but not opioids, countered the antidepressant-like effects of vmPFC electrode insertion, as well as the increased expression of GFAP, TNF-alpha and COX associated with the implants.^104,133^ Another proposed mechanism is the activation of astrocytes (Fig. 2). The insertion of brain electrodes in rodents induces an astrocyte-mediated release of glutamate and adenosine, which modulate the activity of neurons and glial cells in the vicinity of the electrodes.^104,133,146,147^ In cultured astrocytes, the insertion of electrodes activates calcium signalling and adenosine triphosphate (ATP) release.^148,149^

Also important for the development of an insertional effect are the physiological responses of neurons in the region of the implants. As described above, the immediate vicinity of the electrodes is characterized by a reduction in nerve fibre density and the number of cell bodies.^45,96,97^ However, surviving neurons show reduced inhibitory synaptic currents, a downregulation of glutamine synthetase and reduced gamma-aminobutyric acid transmission.^99^ With the chronic interaction between active astrocytes and neurons, the latter tend to become hyperexcitable,^99^ a scenario that may lead to focal and distant changes (Fig. 2).

On a circuitry level, the insertion of electrodes in preclinical models was shown to exert changes at a distance from the target. In rodents, positron emission tomography (PET) studies following vmPFC electrode implantation and DBS showed different metabolic patterns, suggesting the recruitment of alternative networks.^103^ Mice implanted with vmPFC DBS electrodes presented local hippocampal and thalamic volumetric enlargement compared to animals receiving no electrode implants,^105^ an effect attributed to an increase in the size of astrocytes and the diameter of blood vessels.^105^ In AD transgenic mice, the insertion of hippocampal electrodes was found to increase hippocampal neurogenesis and reduce the number of amyloid plaques.^137^

p11 is a protein expressed in brain regions implicated in the pathophysiology of depression^150-152^ that co-localizes with 5-HT_1B_ and other serotonergic receptors.^153,154^ p11 mRNA is reduced in depression states,^155^ and increases following the administration of different classes of drugs and electroconvulsive therapy.^156,157^ This protein has been associated with the antidepressant-like effects of vmPFC electrode implantation.^133^ Favouring the involvement of a serotonergic mechanism in the behavioural consequences of electrode insertion, the administration of para-chlorophenyl-alanine methyl ester, a drug that reduces serotonin synthesis, countered the antidepressant-like response of vmPFC implants in rodents.^133^ In addition, the increase in p11 associated with an insertional effect was mitigated by the administration of anti-inflammatory agents,^104^ suggesting a potential link between neuroinflammation, p11 expression and serotonergic transmission after electrode insertion.

DBS has been shown to reduce inflammatory responses in cultures of astrocytes^143^ and in structures at a distance from the target in rodent models of epilepsy,^158^ stroke,^159^ Alzheimer’s disease,^160^ traumatic brain injury^161^ and parkinsonian states.^142,143^ Similarly, the insertion of STN electrodes mitigated the increased expression of multiple cytokines observed in the striatum of parkinsonian rats, reduced striatal glutamate release and augmented the relative concentration of GABA.^142^

In summary, the insertion of electrodes into the brain is associated with processes that go far beyond a simple mechanical effect. The initial inflammatory response and the subsequent gliotic reaction that occurs near the electrode site induce lasting changes in glial cells and neurons, which may present altered patterns of neurotransmitter release and become hyperexcitable. These combined responses may ultimately induce a complex interplay of focal and distant changes that actively lead to behavioural modifications.

## Insertional effect in clinical practice

**Figure 3:**
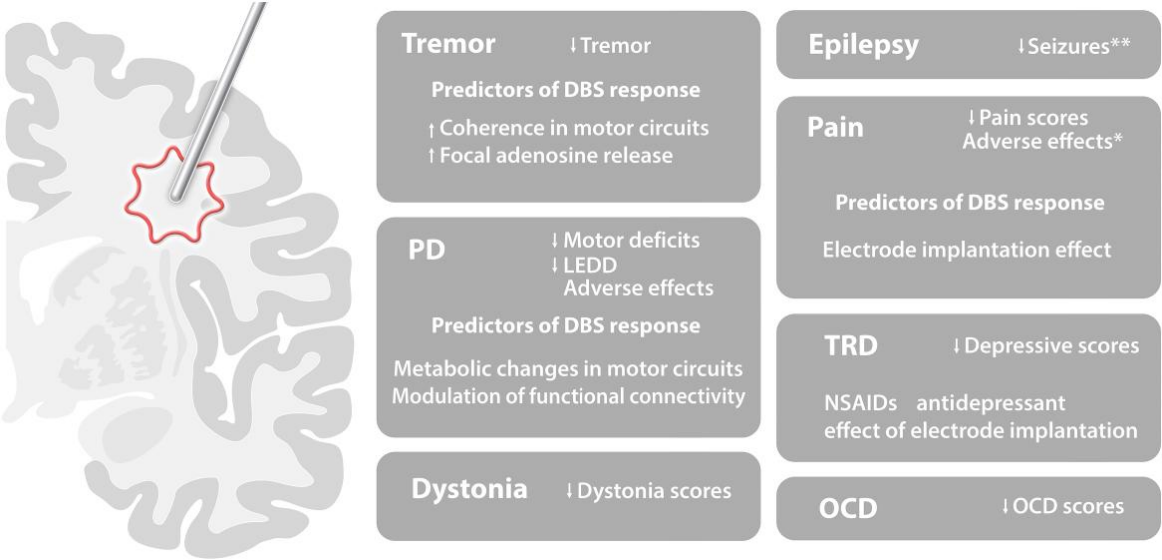
**Clinical consequences of the insertional effect.** NSAID, non-steroidal anti-inflammatory drugs; LEDD, levodopa equivalent daily dosage; PD, Parkinson’s disease; TRD, treatment-resistant depression; OCD, obsessive-compulsive disorder. *Adverse effects of electrode implantation are similar to those of DBS; **controversial results when the hippocampus is targeted.

The amelioration of symptoms following electrode insertion has been reported in multiple conditions. The presence of an insertional effect is more easily demonstrated in patients with movement disorders and epilepsy, as it can be objectively documented. In pain and psychiatry, immediate postoperative improvements are only subjectively reported (Fig. 3).

### Tremor

Some of the first observations of an insertional effect were provided by studies using thalamic (ventral intermediate nucleus; Vim) DBS for Parkinson's disease (PD) and essential tremor (ET).^5-7^ Based on the similarities in outcome between electrode insertion and thalamic lesions (i.e. thalamotomy), the clinical improvement observed after the former was also called ‘microthalamotomy’.

In general, tremor improvement after electrode insertion is more pronounced early after surgery, but may persist for weeks, months and even years.^5,6,162-164^ In ET, but also in PD, series that specifically studied the clinical effects of electrode insertion suggested that this phenomenon was of clinical relevance in ∼50% of patients, with an average duration of 25 days.^162^ In ET, the development of an insertional effect seems to indicate an accurate targeting strategy and predict a good postoperative response to DBS.^163^ In addition to Vim, electrode implants in the region of the zona incerta and prelemniscal radiation have also been associated with an insertional effect, with patients presenting a substantial improvement and even tremor arrest.^165^ In patients with ET and PD, the insertion of electrodes in the posterior subthalamic area was found to reduce the amplitude and duration of saccades.^166^

From a mechanistic standpoint, the insertion of thalamic electrodes in patients with multiple forms of tremor (essential tremor, Parkinson’s disease, multiple sclerosis) decreased coherence between the cerebellar thalamus and electromyography activity at the tremor frequency.^167^ In patients with ET, the implantation of thalamic DBS electrodes increased the focal release of adenosine,^168,169^ corroborating results previously observed in preclinical models.

### Parkinson’s disease

STN electrode insertion in patients with PD can result in a 17–30% reduction in Unified Parkinson’s disease rating scale (UPDRS) scores,^170-174^ and reductions in bradykinesia, tremor and rigidity in the order of 14–42%.^171,172^ Clinical amelioration after electrode implantation in PD is maximal in the first postoperative days but may still be present weeks after the procedure.^175,176^ Similar to what has been described for tremor, PD patients who present an insertional effect were shown to have a good postoperative response to DBS.^177^ Objective improvements associated with electrode insertion have been documented in the finger tapping velocity, pronation-supination, and the boxing with touch tests.^178^

Rarely, negative insertional effects have been observed, including transient dyskinesias in 10% of patients.^171^ Although these are often mild, severe dyskinesias and even ballistic movements have been reported.^179^ One of the most common complications of STN DBS surgery is the development of a decrease in verbal fluency.^180^ This adverse event has also been described following electrode insertion,^181-184^ being attributed to the microlesion of fibre bundles, the cingulum and basal ganglia structures. STN electrode insertion in the postoperative period has also been suggested to alter the recognition of facial emotions,^185^ transiently increase the latency for saccades,^186^ and to induce rapid eye movement sleep.^187^

The development of an insertional effect has also been documented following electrode insertion to the other major target in the treatment of PD—the globus pallidus internus (GPi). One series reported that almost 50% of GPi-implanted subjects experienced a positive insertional effect, with some presenting >50% improvements in UPDRS scores and a reduction in postoperatively LDOPA requirement.^188^ In addition to STN and GPi targets, a substantial improvement in motor symptoms has also been documented following electrode insertion in the prelemniscal radiation, zona incerta (tremor and rigidity),^189^ and in the vicinity of the STN (suboptimally placed electrodes).^190^

In patients undergoing DBS surgery, microelectrode mapping (MER) is a commonly used strategy to refine surgical targeting that has been associated with an insertional effect in some instances.^172,191^ The technique consists of intraoperative recording of local neuronal activity and/or local field potentials along with the administration of low current stimulation.^192-194^ Electrodes used during MER are ∼10-fold thinner than the actual DBS electrodes, but multiple trajectories may be required for an adequate establishment of the optimal target site.^192-194^ Interestingly, positive insertional effects have not been found to correlate with the number of brain penetrations.^172^ The development of an insertional effect following MER correlated with the normalization of the H reflex.^195^

Neuroimaging studies have helped to understand distant effects of electrode insertion. PET studies have shown that the insertion of STN electrodes in PD patients reduces glucose utilization in the putamen, globus pallidus, ventrolateral and mediodorsal thalamic regions, while increasing metabolism in the sensorimotor cortex and cerebellum.^196^ This pattern was similar, but with lower magnitude changes, than the one observed following STN lesions and DBS.^197,198^ An fMRI study involving visually triggered finger tapping has also revealed a lower signal in cortical and subcortical regions (thalamus, basal ganglia) following electrode insertion compared to the preoperative period.^173^ In some of these studies, rigidity and axial symptoms recorded after electrode insertion were inversely correlated to the activation of the putamen and globus pallidus.^173^

In contrast to the somewhat homogeneous results of functional neuroimaging reports, connectivity analyses have shown different patterns of response following DBS and electrode insertion. While STN stimulation induced changes primarily in cortical structures, electrode insertion was associated with an increased centrality and functional connectivity in the brainstem,^199^ as well as a decrease in homogeneity in the default mode network (DMN), prefrontal cortex and the cerebello-thalamocortical circuit.^200^ Also favouring the notion that electrode insertion modulates basal ganglia circuits, electrophysiological recordings have shown a significant increase in low-frequency fluctuations in the putamen and precentral gyrus, a decrease in these fluctuations in structures of the DMN and the executive control network.^174^

Taken together, these findings suggest that the insertional effect is mediated, at least in part, by widespread circuitry modulation. Though the mechanisms responsible for this phenomenon in patients with PD are still unclear, cortical and subcortical oedema,^173^ and the functional or anatomical disruption of white matter bundles in the trajectory of the electrodes have been suggested to play a role.^182^

### Dystonia

Similar to other movement disorders, substantial clinical and objective kinematic improvements have been described in patients with dystonia implanted with GPi electrodes.^178^ In a recent study, almost 80% of patients with Meige syndrome had a 49% reduction in Burke Fahn Marsden Dystonia scores following electrode implantation.^201^ In these patients, a positive correlation was found between the development of an insertional effect and postoperative outcome at 12 months.^201^ In patients with generalized dystonia, trials that reported an insertional effect described a substantial amelioration in dystonic symptoms (often >50%) following GPi electrode implantation lasting days to weeks.^202,203^

### Epilepsy

In epilepsy, patients with distinct seizure types and foci have been treated with DBS in different brain targets.^204^ Anterior thalamic nucleus (ANT) and hippocampus stimulation have been proposed for the treatment of focal seizures, including those originating in the temporal lobe. In contrast, centromedian (CM) and subthalamic DBS have been investigated in patients with generalized seizures.^204^

In the ANT, electrode insertion alone has been shown to substantially reduce the frequency of seizures by 50–70% in more than 50% of patients, an effect that may last from weeks to months.^9,205-208^ In some ANT-DBS studies, the reduction in seizure rate after electrode insertion accounted for most of the benefits observed in the postoperative period, with no further outcome changes being documented after DBS onset.^206^ As for CM electrode implants, a study reported a ≥50% reduction in seizure rate in up to 70% of patients,^209^ an effect that lasted one month following the procedure.^209^ Results in trials implanting hippocampal electrodes have been inconsistent with seizure reduction being demonstrated in one study,^210^ but not in other.^211^

### Pain

Very few reports have described an effect of electrode insertion in chronic pain patients. In one of the few retrospective series addressing this issue, 43% of patients implanted with DBS systems in sensory thalamic regions had a substantial reduction in pain scores (60–100%) following electrode implantation.^8,212^ Recurrence of pain in these patients occurred within a median of 3 months after surgery.^8^ Twice as many patients who presented an insertional effect following electrode placement responded to DBS at long-term.^8^ Similar to Parkinson’s disease, transient side-effects have also been reported after electrode insertion in patients with chronic pain. As an example, a patient with facial pain developed stutter that lasted 12 days following the insertion of a thalamic electrode.^213^

In addition to chronic neuropathic pain, DBS has also been used to treat patients with cluster headache and short-lasting unilateral neuralgiform headache (SUNCT). Brain regions commonly targeted for these applications are the hypothalamus and the ventral tegmental area. A few studies have clearly reported the presence of an insertional effect. In one of the series, two out of 11 patients implanted with hypothalamic electrodes had a substantial clinical improvement 1 month after surgery prior to DBS.^214^ After receiving ventral tegmental area implants, six out of 21 patients with cluster headache^215^ and 4 of 11 patients with SUNCT^216^ had an improvement in the number of attacks after electrode placement that lasted 1–3 months postoperatively.

Another neuromodulation modality under investigation for the treatment of chronic neuropathic pain is motor cortex stimulation. This technique involves the placement of electrodes over motor cortical regions, followed by electrical stimulation delivered through a pulse generator. Despite the promising results observed in open label studies,^212^ trials comparing pain scores during active versus sham treatment^217^ or high versus low stimulation (longer ‘on’ versus ‘off’ stimulation cycling)^218^ found no differences in clinical response. In some of these studies, however, an analgesic effect prior to the delivery of stimulation could be clearly observed.^217^ In a recent report in which the effects of electrode insertion were taken into account, a >50% reduction in pain scores was reported in ∼40% of patients.^219^ More strikingly, the probability of observing some degree of postoperative improvement while receiving stimulation in patients who developed an insertional effect was close to 100%.^219^

### Psychiatry

In psychiatry, DBS has been approved for the treatment of obsessive-compulsive disorder (OCD) and is currently under investigation for conditions such as major depression and post-traumatic stress disorder (PTSD). A meta-analysis of trials on DBS for OCD revealed that the insertion of electrodes into the target in the absence of stimulation (sham) reduced Yale-Brown obsessive-compulsive scale scores by 15%.^220^ In a retrospective chart-review of studies using DBS to treat refractory depression, patients who did and did not receive anti-inflammatory drugs in the postoperative period were separated in groups. Early response during the first week after surgery was similar between groups.^133^ The antidepressant effects of DBS were only observed in patients who did not receive anti-inflammatory drugs.^133^ In a recent clinical trial investigating the effects of DBS in patients with PTSD, one individual (out of a total of four) reported that the best improvement she experienced following surgery occurred in the immediate postoperative period.^221^

## Discussion and perspectives for the design of clinical trials

Though the clinical effects of electrode insertion have been appreciated for decades, it was not until recently that its mechanisms of action and relevance for trial design have been recognized. In humans, an improvement in symptoms following electrode insertion has been documented in almost every DBS application. As described above, this response is maximal within days of the procedure, but may last for weeks and even months. This can be treacherous for the future of the field because, for DBS applications to be approved, a common trial design involves a blinded comparison of active versus sham stimulation (i.e. systems set at zero amplitude or inactive).^222^ Clinical assessments are then commonly conducted at 3 months or 6 months after surgery, with no short-term evaluations. As the development of an insertional effect is rarely considered, its occurrence is not always detected, but can certainly influence the overall results. Without a proper trial design, it is difficult to discern the consequences of electrode insertion from those associated with stimulation-induced plasticity and a carry-over effect from electrode insertion. Ideally, postoperative scores should be serially measured until their values are back to preoperative baseline. At present, improvements in the sham-stimulated group are often attributed to placebo effects, which are certainly a factor to be considered should other factors be discarded.

In theory, should an insertional effect occur, patients in the sham-treated group would have better scores compared to those recorded at baseline and the effects of DBS would be underestimated. For example, even 3 months after surgery, one RCT conducted in patients with PD treated with DBS reported a significantly better quality of life and a modest reduction in motor scores in non-stimulated controls.^223^ In a randomized control trial comparing active versus sham anterior thalamic nucleus stimulation for epilepsy, the frequency of seizures within the first month of electrode placement was in the order of 20% in both groups compared to baseline.^224^ In the sham-treated group, a 20–30% decrease in seizure rate was still observed 3 months after surgery. Only 4 months after the procedure differences between groups became significant.^224^ If the trial had its primary outcome set at 3 months, no differences between active and sham stimulation would have been detected and the study would have likely failed to demonstrate efficacy.

In animal models, several studies have shown that the implantation of vmPFC electrodes induces antidepressant-, anxiolytic- and antianhedonic-like effects.^104,128-130,133-136^ In the clinic, SCG DBS trials have shown a 10–30% reduction in validated depression scores in patients treated with sham SCG stimulation compared to baseline.^10,225^ In a study that initiated SCG DBS soon after surgery, an important reduction in depression scores was noted in 87.5% of patients in the first postoperative week.^226^ This response subsided over the first postoperative month, with only 37.5% of patients being characterized as responders. Only 6 months after stimulation onset a substantial DBS response was recaptured in most patients. This indicates the possibility of an insertional effect immediately after surgery that might have subsided within the first postoperative month.^226^

Similar to animal models, short-term differences between active and sham stimulation in randomized trials are often not significant.^10,225^ Considering that the effect of electrode insertion is so striking in rodents, clinical studies should consider either comparing DBS effects with non-implanted controls, or only finish the sham stimulation timeframe when scores return to baseline levels.

## Conclusions

DBS has become standard of care in the treatment of refractory movement disorders, and is under investigation for the treatment of several neuropsychiatric disorders. In almost all DBS applications, the insertion of electrodes into the target induces some degree of clinical improvement. While this is often more pronounced at short-term, the effect of electrode insertion may last for weeks or even months. Disregarding this phenomenon may lead to potential biases in randomized clinical trials, which may show a symptomatic amelioration in patients with electrodes implanted and an equal degree of improvement following sham or active stimulation.

From a mechanistic standpoint, preclinical work has shown that, in addition to an initial inflammatory response, the placement of electrodes in the brain leads to more protracted responses. These include changes in the physiological interplay between astrocytes and other neural elements, a focal release of gliotransmitters, the presence of hyperexcitable neurons in the vicinity of the electrodes, as well as plasticity and neural changes at a distance from the target. This suggests that, rather than a simple neuroinflammatory response, the placement of brain electrodes leads to changes in neurophysiological substrates and its consequences culminate in a cascade of biological processes. With this in mind, we suggest that the implantation of brain electrodes should be considered to be an active part of the DBS therapy, which includes not only the administration of electrical current but also the placement of electrodes into the desired targets.

## Data Availability

Data sharing is not applicable to this article as no new data were created or analysed in this study.
